# Career choice processes in occupational therapy, speech and language therapy, and physiotherapy: a scoping review

**DOI:** 10.1186/s12909-026-09082-1

**Published:** 2026-04-01

**Authors:** Marina Lange, Nadja Reeck, Christian Kopkow, Bernhard Maria Borgetto

**Affiliations:** 1 ¹HAWK Hildesheim/Holzminden/Göttingen University of Applied Sciences and Arts, Goschentor 1, Hildesheim, 31134 Germany; 2 Department of Therapy Science, Faculty 4, Human Sciences, Brandenburg University of Technology Cottbus-Senftenberg, Senftenberg, 01968 Germany

**Keywords:** Career choice, Allied health professions, Occupational therapy, Physiotherapy, Speech and language therapy, Scoping review

## Abstract

**Background:**

Allied health professions, including occupational therapy, speech and language therapy, and physiotherapy, are integral to modern healthcare systems. Despite their importance, these fields face ongoing challenges in recruiting and retaining qualified professionals. A better understanding of the factors influencing career choice in these professions is essential for developing targeted workforce strategies. This scoping review aims to systematically identify, categorise, and synthesise influencing factors and underlying theoretical frameworks related to career choice processes in these fields, with particular attention to the German context.

**Methods:**

A scoping review was conducted in December 2024 across the databases PubMed, CINAHL, Web of Science, ERIC, PsycINFO, and the Cochrane Library. Eligible sources included quantitative, qualitative, and mixed-methods studies, as well as relevant grey literature in English or German. Two reviewers independently screened and selected studies based on predefined criteria. Data were extracted and thematically analysed using qualitative coding in MAXQDA.

**Results:**

Of 1,852 records screened, 37 studies were included. Thematic analysis identified four main categories of influencing factors: (1) personal factors (demographic characteristics, personality traits, altruism, fields of interest, qualifications, knowledge); (2) social influences (professionals, service users); (3) exposure to the profession (internships and part-time jobs, experience as a patient, media, advisor, events); and (4) professional factors (working conditions, scope of practice). Most studies originated from Australia, the USA, and the UK; none were conducted in Germany.

**Conclusions:**

Career choice in occupational therapy, speech and language therapy, and physiotherapy results from a multifaceted interplay of individual, social, and structural factors. The absence of studies from Germany highlights a substantial research gap and underscores the need for context-specific empirical investigation. These findings offer a foundation for developing evidence-based recruitment and retention strategies and highlight the necessity for future research in the specific context of the German healthcare and educational system.

**Supplementary Information:**

The online version contains supplementary material available at 10.1186/s12909-026-09082-1.

## Background

Allied health professions such as occupational therapy (OT), speech and language therapy (SLT), and physiotherapy (PT) are essential components of modern healthcare systems, contributing significantly to the delivery of evidence-based, patient-centred care [1]. In particular, these professions play a central role in rehabilitation, long-term care, and the management of chronic conditions, especially in the context of demographic change and increasing healthcare complexity [2-4].

The explicit focus on OT, SLT, and PT is justified by their status as key disciplines within physical and cognitive rehabilitation internationally [1, 2] and their shared developmental trajectory within the German context [5-7]. In Germany, they are commonly grouped as core ‘therapy professions’, characterised by similar historical roots in non-academic vocational training and by undergoing comparable structural reforms, including the transition towards academisation and the introduction of direct access models [5-9]. Focusing on these three professions therefore enables a context-sensitive analysis of shared structural conditions while also allowing the identification of profession-specific differences, whereas other allied health fields (e.g., dietetics or podiatry) follow different regulatory structures and educational pathways [5].

Despite their increasing relevance, these professions face persistent challenges in attracting and retaining qualified professionals [10-12]. Workforce shortages have been reported across multiple healthcare systems, reflecting both rising demand and limitations in training capacity and professional attractiveness [4, 12, 13]. In Germany, these challenges are compounded by ongoing structural reforms within the educational system. Since the introduction of model clauses in 2009, which allowed for pilot university-based primary-qualification programmes alongside traditional vocational training, academic pathways for OT, SLT, and PT have been gradually expanded. However, training capacities and the number of graduates remain limited and vary significantly across federal states [5, 6, 8, 9, 14]. For instance, national reports indicate that the proportion of academically trained physiotherapists remains comparatively low (e.g., approximately 2.7% according to recent workforce data), while university enrolment capacities are still restricted [7].

At the same time, a shift in applicant profiles can be observed. Degree programmes increasingly target applicants with university entrance qualifications, while maintaining accessibility for individuals with intermediate school-leaving certificates remains a central challenge [5, 7]. These developments highlight structural tensions within the current educational system and may contribute to ongoing difficulties in recruitment.

In addition, structural factors such as limited professional autonomy, restricted career advancement opportunities, and comparatively low public visibility further influence the attractiveness of these professions [6, 14]. Career decisions in these professions therefore cannot be understood solely as individual choices but must be interpreted within broader structural and institutional contexts.

Understanding how individuals decide to pursue these professions is therefore crucial for developing targeted and sustainable recruitment strategies. Career choice is widely recognised as a complex and multidimensional process shaped by the interplay between individual characteristics, social contexts, and institutional frameworks [15]. Over the past few decades, several theoretical frameworks have been developed to explain these processes, including Social Cognitive Career Theory (SCCT) [16], Holland’s RIASEC model [17], and Gottfredson’s Theory of Circumscription and Compromise [18]. These frameworks emphasise that career decisions emerge from a dynamic interaction between self-efficacy, personal interests, social expectations, and perceived opportunity structures [15].

While extensive research exists for other health professions such as nursing and medicine [19, 21], systematic investigations focusing specifically on OT, SLT, and PT remain comparatively limited. Findings from medicine and nursing cannot be directly transferred to these allied health fields, as they differ substantially in their professional focus, scope of practice, and career structures [14]. OT, SLT, and PT are characterised by rehabilitation-oriented work, long-term therapeutic relationships, and a strong emphasis on functional outcomes, distinguishing them from more acute, medically driven professions [5, 14, 22].

A prior synthesis by Wallis et al. [22] provides an important overview of motivations for entering allied health professions. However, the existing evidence remains largely descriptive and predominantly focuses on Anglo-American contexts. A comprehensive synthesis that systematically integrates influencing factors with underlying theoretical frameworks is still lacking, particularly with regard to how these factors operate across different national contexts.

This gap is especially pronounced in Germany, where systematic empirical evidence on career choice processes in OT, SLT, and PT is virtually absent despite ongoing structural reforms and increasing workforce demands [6, 14]. Without such evidence, it remains unclear how international findings can be transferred to the German context or how recruitment strategies can be effectively tailored to national conditions.

Existing studies suggest that factors such as public recognition, financial considerations, access barriers, and evolving educational pathways may influence career attractiveness [14, 22, 23]. However, an integrated, theoretically informed understanding of how these factors shape career decision-making is still missing.

The application of theoretical frameworks such as SCCT or Holland’s model allows for a more comprehensive understanding of these processes by linking individual motivations with structural conditions. For example, structural barriers may influence self-efficacy beliefs, while social expectations can shape the development of professional interests [16, 24]. Such perspectives are widely considered essential for developing effective interventions in career guidance, educational policy, and workforce planning.

In the German context, where the educational and professional landscape of therapy professions is currently undergoing significant transformation, these insights are particularly relevant. Differentiating between universal motivational factors (e.g., altruism) and context-dependent structural conditions (e.g., training pathways, financial incentives, professional autonomy) may help to develop more targeted and effective recruitment strategies.

This scoping review therefore aims to systematically identify, categorise, and synthesise influencing factors and underlying theoretical frameworks related to career choice processes in OT, SLT, and PT. In addition, the review seeks to integrate these findings within established theoretical frameworks and to identify critical research gaps, particularly with regard to the German healthcare context.

## Methods

### Design and protocol

This scoping review was conducted and reported in accordance with the *PRISMA Extension for Scoping Reviews (PRISMA-ScR)* guidelines [25]. The checklist is provided in Additional File 1. The methodological framework was guided by Arksey and O’Malley [26] and the Joanna Briggs Institute (JBI) recommendations for scoping reviews [27]. The protocol was preregistered on the Open Science Framework (OSF; 10.17605/OSF.IO/T9K6M).

### Review question and eligibility criteria

The review was guided by the Population–Concept–Context (PCC) framework [27] and addressed the following research question:

*What influencing factors and underlying theories*,* models*,* or concepts are reported in the literature to explain career choice processes in occupational therapy*,* speech and language therapy*,* and physiotherapy?*

- Population: Individuals involved in career decision-making processes related to OT, SLT, and PT (including pupils, students, trainees, and graduates).
- Concept: Influencing factors (e.g., motivations, values, socio-demographics, exposures, supports/barriers) and theories/models/concepts explaining career choice processes.
- Context: Educational, clinical, or professional settings; no restrictions by country or health system.

### Inclusion and exclusion criteria

Empirical studies employing quantitative, qualitative, or mixed methods, as well as relevant grey literature (e.g., reports, dissertations), were considered eligible. Publications had to be written in English or German. Exclusion criteria comprised:

- Studies unrelated to OT, SLT, or PT;
- Opinion pieces, editorials, and letters to the editor;
- Inaccessible full texts or records with insufficient data for analysis. The latter was defined as lacking extractable information on influencing factors or theoretical frameworks, or providing only aggregate data that did not allow for a profession-specific analysis of OT, SLT, or PT (e.g., missing detail on specific motives/barriers or unclear participant characteristics).

### Search strategy

A comprehensive literature search was conducted in December 2024 across the following databases: PubMed, CINAHL, Web of Science, ERIC, PsycINFO, and the Cochrane Library. The search strategy combined keywords and subject headings that aligned with the PCC framework. No country filters were applied to avoid the exclusion of potentially relevant studies. To enhance the capture of evidence from Germany, we complemented the database searches with targeted German-language grey literature searches across relevant institutional repositories and professional association websites. The complete search strategy is available in Additional File 2.

### Study selection and screening

The search results were imported into CADIMA, a web-based tool for systematic reviews, where duplicates were removed [28]. Two reviewers (ML and NR) independently screened titles and abstracts against the eligibility criteria. Prior to the main screening, the reviewers piloted the inclusion and exclusion criteria on a random sample of 25 publications to ensure consistent application. If agreement was below 75%, discrepancies were discussed to identify ambiguities in the application of the eligibility criteria. Subsequently, the operational definitions and decision rules for applying the inclusion and exclusion criteria were refined to ensure consistent interpretation before proceeding with the main screening [29]. Any disagreements during the screening process were resolved through discussion until consensus was reached.

Full texts of potentially relevant studies were retrieved and assessed for eligibility by both reviewers, with reasons for exclusion documented at each stage [29]. Reference lists of included studies were additionally screened to identify further relevant articles (ML).

Only studies published from 2005 onwards were included at the full-text stage. This cut-off was chosen to reflect recent developments in the education, regulation, and professionalisation of allied health professions. Since the mid-2000s, many countries, including Germany, have implemented substantial reforms, such as the academisation of OT, SLT, and PT, as well as the introduction of degree-based qualification pathways [6, 8, 14]. These developments are often aligned with the Bologna Process, which aims to enhance the comparability and compatibility of European higher education systems through a three-cycle structure (Bachelor/Master/Doctorate) and instruments such as ECTS, quality assurance, and the Diploma Supplement [30]. They have significantly influenced curricula, admission frameworks, professional roles, and career pathways across the European Higher Education Area. To ensure that all relevant literature reflecting these diverse and evolving contexts was captured, a broad initial search was conducted without a time limit. This strategy enabled the inclusion of earlier seminal literature during the title and abstract screening phase to inform terminology and theoretical framing, while avoiding the premature exclusion of conceptually relevant records. Consistent with the methodological framework for scoping reviews, which recommends maximising sensitivity, the time limit was applied only at the full-text stage [26, 29].

### Data extraction

A standardised data extraction template was developed (Additional File 3). One reviewer (ML) extracted relevant data from the included studies, and a second reviewer (NR) verified the extracted data for accuracy and completeness. The level of agreement during this verification process was very high (> 95%). Discrepancies were resolved through discussion and consensus. Extracted information included study characteristics (article ID, title, author(s)/year, country, study aim/purpose, population, study type, method of data collection, results) and quotations related to factors influencing career choice.

### Analysis and presentation of results

The extracted data were imported into MAXQDA 24 (VERBI Software, Version 24.8.0) for thematic analysis. An inductive coding approach was used to categorise the factors influencing career choices. This approach was chosen to enable a comprehensive and unbiased mapping of the evidence, which is consistent with the exploratory nature of scoping reviews. It allowed for the identification of all reported influencing factors, including those potentially specific to the therapy professions, without constraining the analysis to pre-defined theoretical frameworks. Given that many of the included studies did not explicitly apply established career choice theories, an inductive approach ensured that relevant data were not overlooked or forced into existing models. The resulting empirical categories were subsequently mapped onto established career choice theories in the discussion to evaluate their conceptual alignment [31]. Initially, two reviewers (ML and NR) independently coded a subset of the data (30%). Inter-rater agreement for this pilot double-coding was substantial (Po = 0.79; κ = 0.78). The coding results were then compared, discussed, and consolidated into a preliminary coding system, which refined, merged, or expanded existing codes as necessary. This coding system remained adaptable, allowing for modifications if new relevant categories emerged during the subsequent coding process. To ensure reflexivity and track analytical decisions, memoing was utilized throughout the coding process. Furthermore, regular peer debriefing sessions between the reviewers served to critically reflect on the emerging coding system and mitigate potential individual biases. Once consensus was reached, both reviewers applied the unified coding system to the entire dataset. Following this step, the categories were re-evaluated and adjusted as needed, resulting in a finalised coding framework. The extracted factors influencing career choices could be categorised into more than one code. Additionally, one reviewer (ML) separately coded the data by profession (OT, SLT, PT) to allow for profession-specific analyses. Any discrepancies in coding were resolved through discussion to ensure consistency and reliability. The coded factors were subsequently grouped into broader thematic categories. The distribution of coded factors was analysed to identify overarching themes, which were then mapped to the review objectives.

## Results

### Search results

**Fig. 1:**
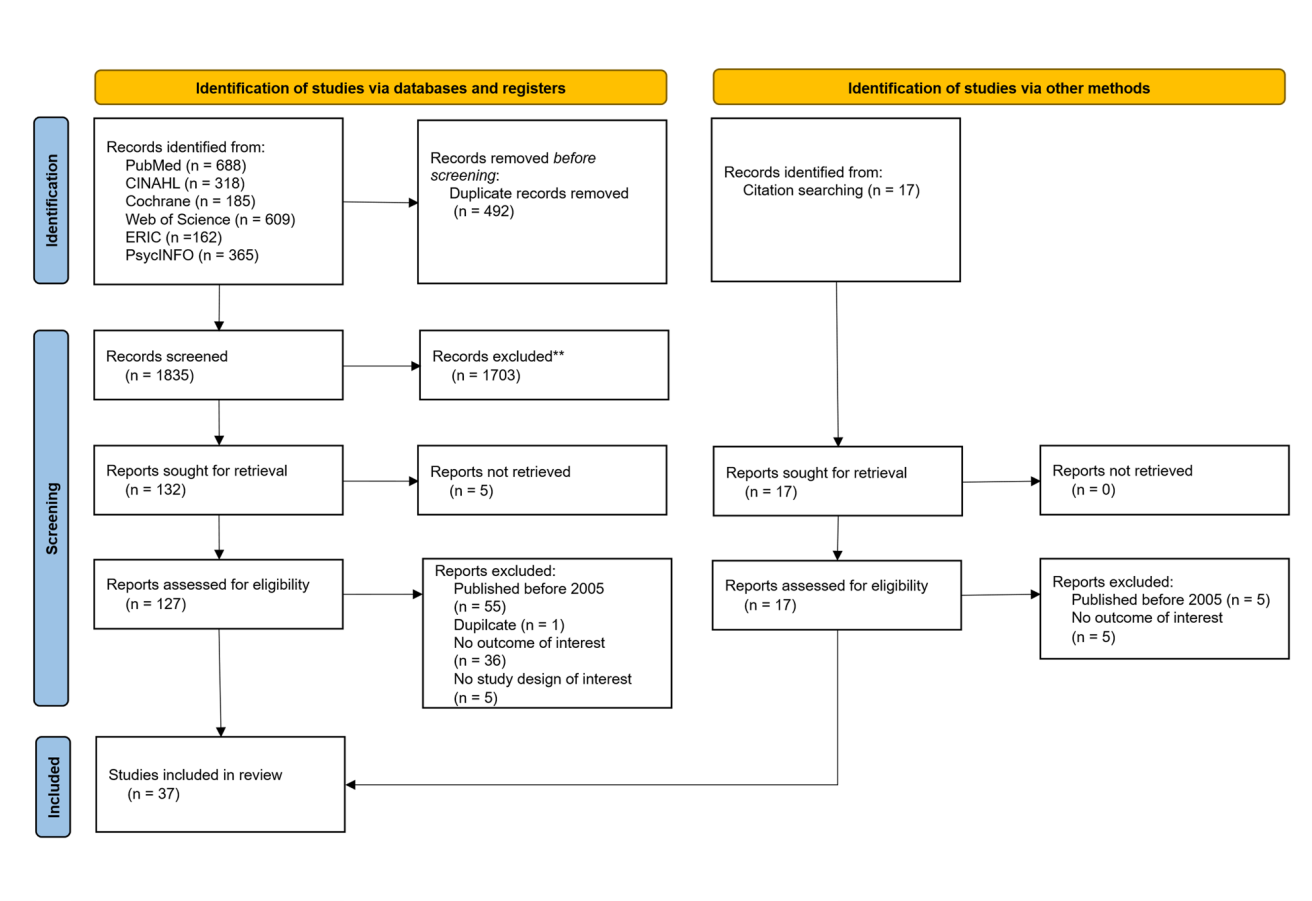
Selection of sources of evidence. From: Page MJ, McKenzie JE, Bossuyt PM, Boutron I, Hoffmann TC, Mulrow CD, et al. The PRISMA 2020 statement: an updated guideline for reporting systematic reviews. BMJ 2021;372:n71. doi: 10.1136/bmj.n71

The initial database search identified 2,344 records. After removing 492 duplicates, 1,852 titles and abstracts were screened. Of these, 149 full-text articles were assessed for eligibility, and 37 studies met the inclusion criteria. A detailed overview of the selection process is provided in the PRISMA flow diagram (Fig. 1).

### Characteristics of included sources of evidence

Of the 37 included studies [32-68], 20 employed quantitative methods [33, 35, 38-41, 43, 46-48, 51, 53, 54, 57, 59, 60, 62, 65, 66, 68], 10 were qualitative studies [32, 34, 36, 44, 45, 50, 52, 55, 56, 67], and 7 used a mixed-methods approach [37, 42, 49, 58, 61, 63, 64]. The publication years ranged from 2005 to 2024, with sample sizes varying between 1 and 941 participants.

The included studies focused on allied health professions, with 21 examining factors influencing career choice in PT [32, 33, 35, 36, 38, 39, 41-43, 46-48, 50, 52, 53, 55, 57, 61, 63, 65, 68], 17 in OT [32, 33, 35, 37, 40-45, 51, 53, 60-62, 66, 67], and 12 in SLT [32, 34, 41, 43, 49, 54, 56, 58-60, 64]. Several studies investigated factors across two or all three of these professions simultaneously [32, 33, 35, 41-43, 53, 60, 61]. The research originated from a variety of countries, with the largest number conducted in Australia, the USA, and the UK. No studies from Germany were included. A detailed summary of the characteristics of the included studies is provided in Additional File 4.

### Review findings

#### Use of theoretical frameworks

A key aspect of the research question was to identify theories, models, or conceptual frameworks used in the literature to explain career choice processes. Of the 37 included studies, only four explicitly anchored their design, analysis, or interpretation in an established theory or conceptual model. These included the Systems Theory Framework [34], Bandura’s self-efficacy, applied as a theory to guide program design and evaluation [46] and as a conceptual lens for interpreting identity trajectories [44], and anticipatory socialization and professional identity theories [50]. The remaining 33 studies were primarily descriptive or exploratory, focusing on empirically identifying isolated motives and barriers without theoretical grounding. Consequently, the extracted data predominantly yielded empirical influencing factors, which formed the basis for the subsequent thematic analysis.

Thematic analysis generated a total of 418 codes (Table 1). Of these, 368 represented influencing factors related to career choice, while the remaining 50 were descriptive, capturing frequency distributions across the three professions. These influencing factors were categorised into four main categories: (1) Personal Factors, (2) Social Influences, (3) Exposure to the Profession, and (4) Professional Factors. The contents and thematic focus of these categories are detailed below.

**Table 1:** Overview of the coding system, frequency of codes and representative quotations

Category and Subcategory	Frequency	Quotations
Total Codes	418	
Total Influencing Factors	368	
1. Personal Factors	138	
Demographic characteristics	20	“The status of a profession […] seemed to be important to students and clinicians of South Asian heritage in relation to their families: “I think some of it comes down to status, ‘cause parents are always saying things like ‘oh well you don’t want to do this’…” [55]
Personality traits	10	“Maeve also decided that a physiotherapy career suited her as she was aiming to do something “more interactive” and “physically moving” rather than “sitting at a desk job”.” [50]
Altruism	28	“Laci expressed that she worked through speech issues with a speech-language pathologist as a child and reflected, “As I’ve gotten older, I’ve just wanted to help other people, like she helped me.” [32]
Fields of interest	35	“Many students identified that an interest in topic areas relevant to speech pathology were influential, particularly the atypical combination of English and science-based subjects.” [34]
Qualifications	20	“[…] physiotherapy was my second option because I wanted dentistry but […] since I couldn’t do dentistry, I chose physiotherapy […]” [52]
Knowledge	23	“I had never even heard of a speech pathologist, didn’t have a clue and it was just so interesting to watch […]” [34]
Other Personal Factors	2	“[…] career that is flexible enough to allow for a career break to have a family.” [56]
2. Social Influences	61	
Professionals	22	“Respondents reported being influenced by their fathers, mothers, siblings, or by family members of close friends if they had a career as a physical therapist and that having a significant impact on their understanding and views of the profession.” [38]
Service users	11	“Participants said, for example: ‘my cousins, who I am really close to, had a lot of speech therapy for years. I just found it so appealing’ and ‘my sister went to a speech pathologist’ and ‘a friend’s little brother had speech therapy’.” [34]
General social influences	28	“In fact, the influence of relatives is seen as a factor […] As one careers advisor (CA6) explains: I’ve had lots of young people where they’ve been ‘oh, my mum wants me to do this’ or ‘dad said this’ and I think that’s a big influence on them.” [56]
3. Exposure to the Profession	60	
Internships and part-time jobs	10	“Jolien got to experience occupational therapy for the first time during a mandatory placement in high school. She did a placement in a nursing home and had to follow every discipline. There she came in contact with an occupational therapist. […] I have found that extremely meaningful.” [44]
Experience as a patient	18	“She was inspired to become a physical therapist after experiencing an injury while cheering in high school and receiving care from a physical therapist. She said, “He explained everything and it was just like, at that moment I was like, I want to make a difference like this.” [32]
Media	7	“Sofie learned about occupational therapy when she came across the word on the internet. She was intrigued and searched for more information.” [44]
Advisors	10	“Other respondents described having a […] mentor suggest physical therapy because they saw it as a compatible career for them.” [38]
Events	6	“Three interviewees came in contact with occupational therapy at the higher education fair (SID-in) … it was the information booth of occupational therapy, which I had never heard of before.” [44]
Other forms of exposure	9	“The majority of students who had professional exposure to occupational therapy identified that it was the most influential factor in their career choice.” [62]
4. Professional Factors	109	
Working conditions	46	“[T]he distribution of factors influencing the students to not continue physiotherapy as a career. […] Low salary, no professional autonomy, not professionally recognised and not respected by the multidisciplinary team is tied as the second most influential factors (31.25%).” [68]
Scope of practice	58	“Another attractive element for the profession was the diversity of tasks or the variety of work, as this occupational therapy student illustrated: There are many different things you can do. And then, I like it when things move, when there are new challenges.” [67]
Overall Career Perceptions	5	“On the pragmatic side, they described how physiotherapy would be a satisfactory career based on their perception of opportunity to choose their type of practice and have day-to-day flexibility along with financial stability.” [50]
Professions Mentioned (Descriptive)	50	
Occupational Therapy	17	
Speech and Language Therapy	12	
Physiotherapy	21	

#### Personal factors

This category encompasses individual characteristics, motivations, and competencies influencing career choice in allied health professions. It includes the subcategories demographic characteristics, personality traits (including altruism, which was conceptualised as a subcategory of personality traits), fields of interest, qualifications, and knowledge.

##### Demographic characteristics

Gender, ethnicity, socioeconomic status, and age substantially shaped perceptions of and preferences for these health professions. Gender-related stereotypes contributed to the perception of SLT as “female profession”, potentially limiting their attractiveness to male students and impacting their social status [49, 56, 63]. Socioeconomic background also played a crucial role, with students from higher-income households and with parents with higher educational attainment being disproportionately represented in therapy professions [40, 49, 52, 53]. In contrast, individuals from ethnic minority backgrounds often lacked access to role models in healthcare [49, 63]. Cultural norms also shaped perceptions of professional prestige, for example, medicine and pharmacy were frequently perceived as more prestigious than OT, SLT, or PT in certain cultural contexts [52, 55, 63]. Career choices in the allied health professions were not limited to young adults entering their first occupation. Several studies highlighted the significance of conscious career reorientation later in life, with some individuals choosing a therapy profession as mature students. Such decisions were often motivated by accumulated life experience, a desire for more meaningful work, or dissatisfaction with a previous career path [50, 56, 59, 67].

##### Personality traits

Students’ decisions to enter allied health professions were frequently aligned with specific personality traits valued in healthcare contexts. Based primarily on self-reported personality inventories and qualitative self-descriptions, students across the professions frequently characterised themselves as helpful, cooperative, and self-directed. Key traits mentioned include conscientiousness, openness to new experiences, and emotional stability [36, 43, 59]. Students pursuing OT were often described as patient and empathetic [43, 66, 67], those choosing SLT exhibited strong communication and organisational skills [43, 56, 59] while students in PT frequently identified with an active, sport-oriented self-image [36, 38, 43, 47, 50, 63].

##### Altruism

The desire to help others was a central motivation across all three professions [32, 34, 36, 38, 42, 50, 53, 54, 57, 66-68]. This altruistic orientation was expressed in diverse ways, ranging from a preference for direct patient interaction to a broader emphasis on societal wellbeing [42, 53, 66-68]. While altruism was universally highly rated, some studies indicated nuanced and context-dependent gender differences rather than stark contrasts. For example, in certain settings, women tended to place a slightly stronger emphasis on the direct therapeutic relationship and the visible progress of individual clients [42, 46]. Men, while also highly valuing the aspect of helping others as an intrinsic motivation, were occasionally found to weigh this alongside tangible professional outcomes, such as career advancement or financial status [42, 47, 56, 57]. However, given the small effect sizes and the variability across different professions and cultural contexts, these variations should be interpreted cautiously.

##### Fields of interest

Many students reported a strong interest in health-related topics, science, social interaction, or movement as central to their decision-making [32, 34, 38, 45, 47, 48, 50, 54, 64, 66-68]. A close connection to sports and movement was common in PT [38, 42, 47, 50, 57, 68], an interest cited particularly by male students [38, 47, 50], whereas an interest in language and sciences was characteristic of SLT [34, 42, 54, 56, 58, 64]. Creative or person-centred interests were prominent in OT [32, 42, 45, 66, 67]. The integration of medical knowledge with practical, social, or creative elements was commonly described as particularly appealing [32, 38, 64, 67].

##### Qualifications

Academic qualifications shaped career pathways in two distinct ways. On the one hand, high academic achievement was a prerequisite for competitive programs, such as physiotherapy, which tended to attract a large proportion of high-performing students [33, 35, 65]. On the other hand, for some individuals, the decision to pursue a therapy profession was a direct consequence of not meeting the even higher admission criteria for their first-choice careers, such as medicine or dentistry, positioning the allied health professions as a highly valued ‘second choice’ [35, 52, 67]. Beyond these objective grades, career decisions were also influenced by students’ personal perceptions of their own capabilities, such as confidence in their academic skills (academic self-efficacy) and a sense of being well-suited to the demands of a healthcare profession [36, 38, 49].

##### Knowledge

Awareness of OT, SLT, and PT among prospective students and the general public was generally low, particularly when compared with medicine or nursing. Many students reported little understanding of the professions prior to their career exploration [34, 37, 44, 49, 55, 61, 63]. The perception of these fields as academic degree programmes was also low, especially among minority ethnic groups [49, 55, 61, 63]. OT was perceived as less well-known [44, 61], while SLT was seen as less scientifically grounded and as a female-dominated field [49, 56, 63]. Noticeable gender-specific and cultural differences were apparent. Men were less familiar with the SLT profession [49], and in some cultural contexts, PT was stereotypically equated with massage or non-professional roles [55, 63]. Furthermore, perceptions of academic status, income potential, and social prestige, often based on misconceptions or insufficient information, also influenced the attractiveness of the professions [49, 55].

#### Social influences

This category emphasises the central role of the social environment. Interpersonal influences were a key driver in decision-making, manifesting in various forms: from direct advice and suggestions provided by family and friends, to the influence of healthcare professionals as role models, and the vicarious impact of observing therapy experiences within one’s social circle [32, 34, 36, 38, 40-42, 44, 47, 49, 52, 53, 55, 56, 62-64, 68].

##### Professionals

Healthcare professionals often act as role models, shaping the perceptions of prospective students through their experiences and professional attitudes. Parents working in healthcare were particularly influential, providing a realistic picture of professional demands [32, 34, 38, 40, 41, 49, 53, 56, 62, 67].

##### Service users

Indirect personal exposure to therapy, most often through family members or close friends who have themselves received therapy, can exert a significant impact on career choice. Positive reports about the effectiveness of therapy and meaningful relationships with therapists can spark interest in pursuing an allied health profession. Conversely, negative impressions or stereotypical views may create uncertainty and deter potential applicants [32, 34, 41, 49, 56].

#### Exposure to the profession

In contrast to the influence of individuals within one’s social network, this category focuses on direct, experiential contact with professional activities and the work environments themselves. These experiences were found to contribute significantly to career decision-making by providing realistic insights into daily tasks and demands. Early professional exposure was associated with a higher likelihood of considering a career in OT, SLT or PT [32, 34, 36-38, 41, 44, 53, 56, 61, 62, 67, 68], with various forms of direct contact reported as influential.

##### Internships and part-time jobs

Practical experiences, such as school internships, volunteer services, or part-time employment provide direct insight into the professional environment. These opportunities enable individuals to align their theoretical assumptions with the practical realities of the profession, thereby fostering a more realistic understanding of the profession [32, 38, 41, 44, 67].

##### Experience as a patient

Personal experience as a patient was identified as a significant influencing factor. Direct contact with therapists during one’s own treatment provided immediate insight into their professional role. Positive experiences with therapy often fostered intrinsic motivation to pursue a career in the field [32, 38, 50, 52, 56, 61].

##### Media

Digital and print media, particularly the internet, professional publications, and university guides, served as important resources. These formal sources of information often supplemented personal experiences and were used to deepen interest or initiate engagement with a potential career path [34, 36, 38, 41, 44, 68].

##### Advisors

Career and guidance counsellors, teachers, coaches, and mentors were identified as influential individuals who could encourage consideration of a health profession through direct recommendations or informal conversations [32, 37, 38, 41, 44, 51, 53, 67, 68].

##### Events

Career fairs, university open days, and other subject-specific events provided structured opportunities to engage with health professionals, direct interaction and hands-on experiences through interactive formats [41, 44, 46].

#### Professional factors

This category includes structural and content-related features of health professions that significantly influence their attractiveness.

##### Working conditions

Financial aspects, job security, and career advancement opportunities were frequently identified as central criteria for career decisions. While these aspects were generally important to all students, some evidence points to subtle gender nuances, with male participants occasionally placing a slightly higher emphasis on economic factors and status, depending on the specific professional and cultural context [47, 56, 57]. A stable job market, the potential for a reliable income, and good career prospects were highlighted as positive attributes. The flexibility of work models, including different employment types and options for self-employment, was also seen as advantageous [32, 38, 39, 45, 47, 48, 50, 53, 56, 57, 59, 60, 65, 68]. However, limitations such as restricted professional autonomy and lack of parity within interdisciplinary teams were perceived as barriers to long-term job satisfaction [52, 63, 68].

##### Scope of practice

The diversity of tasks and the potential for creativity within daily practice were important factors contributing to the attractiveness of these professions [32, 34, 50, 67]. Opportunities to work with various patient groups, to develop individualised therapy concepts, and to establish meaningful therapeutic relationships were described as particularly rewarding [32, 44, 50, 66, 67]. However, stereotypical perceptions persisted [49, 56]. For example, PT was often equated solely with sports-related activities, potentially leading to misunderstandings during initial career consideration [38, 47, 50, 63].

**Fig. 2:**
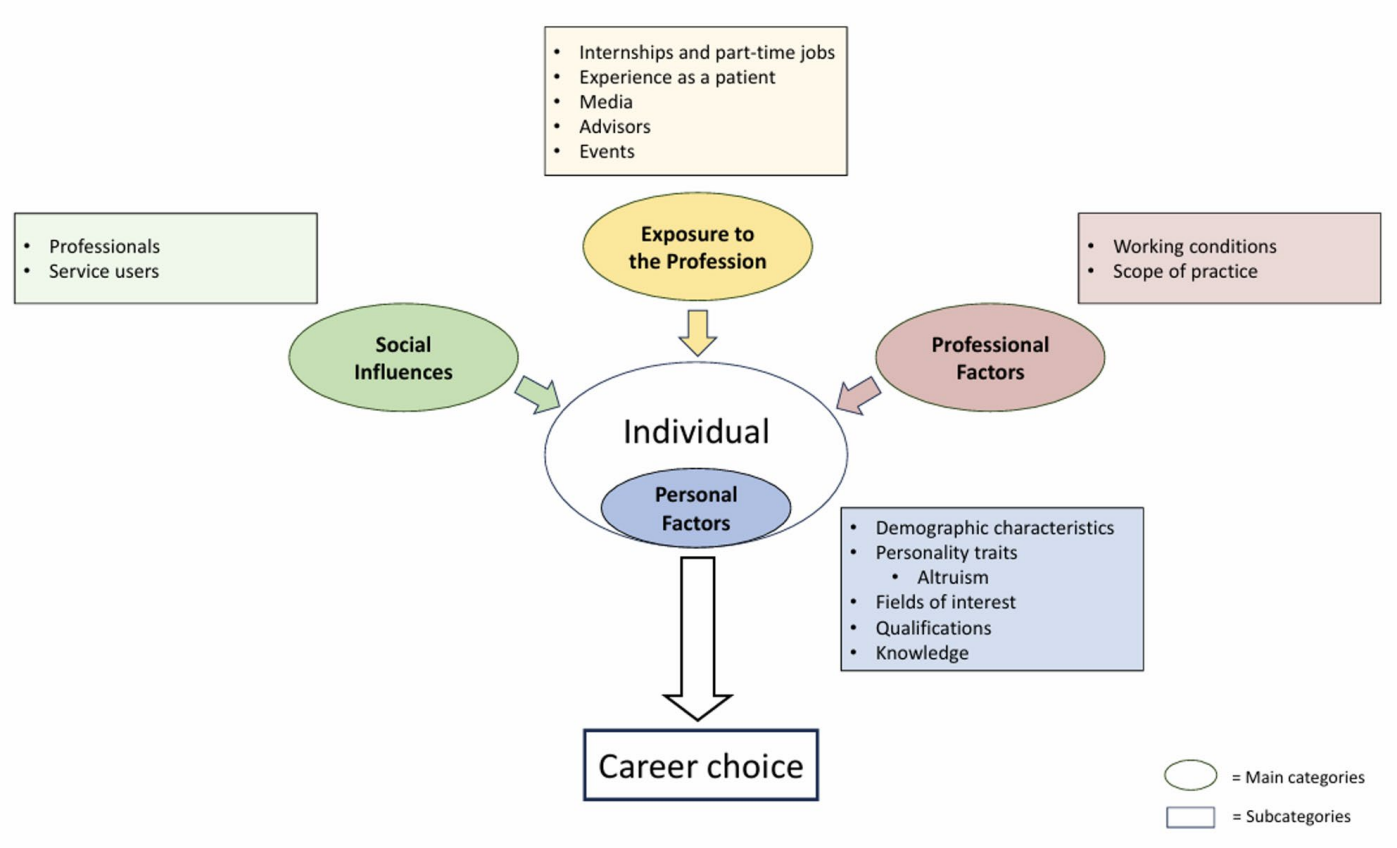
Conceptual model of the factors influencing career choice in the allied health professions of OT, SLT, and PT

A conceptual summary of the four main categories and their respective subcategories is illustrated in Fig. 2.

## Discussion

The aim of this scoping review was to systematically identify, categorise, and synthesise influencing factors and underlying theoretical frameworks related to career choice processes in OT, SLT, and PT. Based on the analysis of the literature, a framework of four main categories was developed, illustrating the multifaceted nature of these influencing factors. These categories, “Personal Factors”, “Social Influences”, “Exposure to the Profession”, and “Professional Factors”, provide distinct perspectives on the decision-making process. As illustrated in the conceptual summary presented in the results (Fig. 2), these findings depict career choice as a multidimensional process where a diverse array of personal, social, and structural factors converge to shape an individual’s decision. The category of “Personal Factors” focuses on the individual attributes and biographical backgrounds that shape a person’s engagement with health professions. “Social Influences” describe how the social environment moulds career choices, while “Exposure to the Profession” covers the direct personal experiences that allow for a reality-based assessment. Finally, “Professional Factors” captures the structural and content-related characteristics of the professions themselves.

This review represents, to our knowledge, the first systematic synthesis of influencing factors across these three health professions. A thorough understanding of these factors is essential for designing effective measures for career guidance and recruitment. However, despite a comprehensive analysis, research gaps remain, particularly concerning long-term career trajectories and the impact of specific support interventions.

### Personal factors in the context of career choice theories

The findings regarding personal factors, such as the prominence of altruism and specific interests, align strongly with the principles of Social Cognitive Career Theory (SCCT) [16], which identifies self-efficacy, outcome expectations, interests, and personal goals as central variables in career decisions. This review confirms the theory’s relevance, showing that a distinct motivational profile, characterised by an intrinsic desire to help others and a strong interest in health or social topics, is a key driver for individuals choosing these professions [34, 38, 45, 53, 67]. Furthermore, the identified importance of a realistic understanding of the profession directly relates to SCCT’s emphasis on developing differentiated outcome expectations and a vocational self-concept [37, 38, 61].

In this context, Betz [24] emphasises that self-efficacy is particularly relevant in gendered career decision-making, as it influences whether individuals perceive themselves as capable of succeeding in fields that deviate from traditional gender roles. Building on this concept of the self, Gottfredson’s Theory of Circumscription and Compromise [69] further explains how societal expectations and gender norms shape which professions are perceived as acceptable or attainable. According to this model, individuals tend to eliminate certain careers early on if they conflict with their developing self-concept or socially prescribed roles. A strong perceived fit between one’s vocational self-concept and the ethos of a profession is thus a key prerequisite for a stable and confident career choice.

Further studies support this finding, showing that demographic characteristics, particularly gender, significantly influence whether a profession is perceived as “typically female” or “gender-neutral,” which in turn affects both subjective fit and societal acceptance [70-72]. Moreover, cultural and societal frameworks substantially shaped the perception and evaluation of health professions. Depending on the social context, certain occupations are regarded as particularly prestigious or, conversely, as less suitable for specific groups [73]. These findings underscore that career choice processes are not purely individual but are deeply embedded in and constrained by broader social, cultural, and cognitive frameworks.

### Social influences: a social cognitive and social learning perspective

The significant impact of social influences found in this review strongly supports their central role within SCCT, where social contexts are considered crucial for the development of self-efficacy and outcome expectations [16]. These influences are deeply embedded in an individual’s social environment and are significantly shaped by interactions with family, friends, and professionals [74]. Such relationships provide essential informational, emotional, and motivational support throughout the career decision-making process. Discussions with practicing professionals are particularly impactful, as they offer authentic insights into the daily realities of a profession and foster a more nuanced understanding of its demands and opportunities. Additionally, experiences shared by individuals who have received therapeutic services offer complementary, often emotionally salient, perspectives that further shape how allied health professions are perceived.

Krumboltz’s Social Learning Theory of Career Decision Making [75] offers a detailed framework for these mechanisms, emphasising the role of observational learning and social reinforcement. This review identified two primary forms of such modelling: the influence of professionals acting as role models, and the vicarious impact of observing the therapy experiences of service users within one’s social circle. These observations contribute to vocational preferences by shaping beliefs about what career paths appear attainable, desirable, and appropriate. Modelling processes are particularly effective when the observer perceives similarity to the model, whether in terms of gender, background, or personal values [76]. This may explain why supportive family contexts and visible, relatable role models are especially critical for women and underrepresented minorities navigating their career choices. In such cases, social support and identification with successful others can enhance confidence in one’s capabilities and widen perceived career options [77].

### The role of direct experience in shaping career cognitions

The findings on direct professional exposure provide strong empirical support for its role as a key mechanism in developing self-efficacy and outcome expectations. Through authentic learning experiences, such as internships, and part-time or volunteer work, individuals gain insight into the professional environment and can test their abilities in real-world settings. Successfully mastering tasks and receiving constructive feedback during these experiences enhances confidence in one’s own abilities (self-efficacy) and fosters more realistic and differentiated expectations about the nature, demands, and rewards of the profession [24, 70, 71]. These cognitive appraisals shape whether individuals view certain career options as attractive, achievable, and worthwhile.

This mechanism is further supported by Krumboltz’s Social Learning Theory of Career Decision Making [75], which emphasises learning through observation and direct experience. First-hand engagement with the profession, including exposure to vocational challenges and interactions with professionals, contributes to the refinement of career aspirations through reflection and social reinforcement. In particular, school-based career programs, informational events, and structured guidance initiatives offer opportunities for vicarious learning, guided discussion, and active exploration [78].

Additionally, these findings align with Holland’s Person-Environment Fit Theory [17], according to which career decisions are optimised when individuals perceive a strong congruence between their personal characteristics and the demands and values of a given profession. Direct contact enables this type of evaluation and increases the likelihood of a well-informed, sustainable career choice.

A notable and often underestimated form of such exposure is the personal experience of being a patient [32, 38, 50, 52, 56, 61]. Several studies included in this review found that individuals who had previously undergone therapy themselves reported a greater interest in entering the health professions, often based on a desire to “give back” or replicate a positive experience. This unique perspective provides an emotional and experiential dimension to professional orientation and may serve as a motivational catalyst, especially in the context of health and helping professions [79].

### Perceptions of professional factors as environmental supports and barriers

The perception of professional factors aligns with the concepts of environmental supports and barriers in Social Cognitive Career Theory (SCCT) [16, 80]. Such contextual conditions influence self-efficacy, outcome expectations, and ultimately motivation to enter or remain in a profession [24, 70]. In this review, factors perceived as supports include stable employment prospects, diverse fields of activity, flexible work models, opportunities for professional autonomy, and interprofessional collaboration [32, 34, 38, 39, 45, 47, 48, 50, 52, 53, 56, 57, 59, 60, 63, 65, 67, 68]. These aspects are commonly associated with positive outcome expectations, such as achieving financial stability, long-term job satisfaction, or a meaningful contribution to society [81]. Favourable perceptions of structural conditions can foster confidence in achieving one’s professional goals, thus reinforcing interest and commitment.

Conversely, factors perceived as barriers include limited career advancement opportunities, high workload, emotional strain, insufficient remuneration, time pressure, and a lack of societal and interdisciplinary recognition [32, 34, 38, 39, 45, 47, 48, 50, 52, 53, 56, 57, 59, 60, 63, 65, 67, 68]. These negative perceptions may undermine confidence in professional success, lead to diminished outcome expectations, and function as deterrents in career decision-making. Particularly in professions such as OT, SLT, and PT, the interplay of emotional demands and limited external appreciation can result in ambivalence toward retention and career persistence [52, 63, 68].

These findings are consistent with Holland’s Person-Environment Fit Theory [17], which posits that satisfaction and persistence in a career depend on the degree of congruence between personal values, interests, and the environmental conditions of a profession. A high perceived “fit” may buffer against professional stressors and strengthen the willingness to remain in the field. Conversely, a perceived “lack of fit”, for example, due to poor recognition or limited autonomy, can lead to dissatisfaction, withdrawal, or career change.

Ultimately, the decision-making process involves a pragmatic evaluation in which individuals weigh perceived supports against anticipated barriers to determine whether a profession offers a viable and rewarding long-term path. Thus, the interplay between structural realities and cognitive appraisals, as highlighted by SCCT, is a key determinant of career intentions in the allied health professions [16, 24, 70, 80, 81].

### Convergence and contrast across the professions: implications for targeted recruitment

Building on these findings, a comparative synthesis across the three professions provides further insight into both shared and distinct patterns. Importantly, the results reveal both substantial convergence and meaningful divergence across the three professions. Across all fields, intrinsic motivations, particularly altruistic values and an interest in health-related topics, emerge as core drivers [32, 42, 53]. This shared motivational profile reflects the fundamentally person-centred orientation of allied health professions [43]. In addition, social influences and opportunities for direct exposure consistently function as key mechanisms through which career preferences are formed and stabilised across the board [38, 49, 62]. This convergence suggests that career choice in these professions is less determined by fundamentally different value systems, but rather by how similar core motivations are channelled into distinct professional identities [17, 18].

At the same time, the analysis points to distinct profession-specific patterns. Physiotherapy is more frequently associated with interests in physical activity, rehabilitation, and sports-related contexts, often attracting individuals with a strong orientation towards movement and physical performance [38, 47, 50]. In contrast, occupational therapy tends to appeal to individuals who value holistic, client-centred approaches and the promotion of participation in daily life, often emphasising creativity and adaptability [32, 45, 67]. Speech and language therapy, by comparison, is more strongly linked to interests in communication, language, and cognitive processes, frequently attracting individuals with a focus on interpersonal interaction and developmental or neurological aspects of care [34, 54, 58]. These differences may reflect not only variations in task profiles, but also distinct professional narratives and public images, which shape how potential applicants perceive “fit” with a given profession.

A key contribution of this review lies in its implications for recruitment and workforce development. The identified similarities across professions indicate that certain strategies may be broadly effective, such as providing early and authentic exposure to professional practice and strengthening career guidance [62, 82]. However, the observed differences highlight the need for more differentiated and targeted approaches. Recruitment strategies must align communication and outreach activities with the specific motivational profiles associated with each profession [10, 82]. For example, emphasising physical activity and rehabilitation contexts may be particularly effective in attracting candidates to physiotherapy [83] whereas highlighting creativity, participation, and holistic care may resonate more strongly with potential occupational therapists [10]. This also implies that generic recruitment campaigns risk overlooking profession-specific drivers and may therefore be less effective in addressing workforce shortages, as recruitment and retention in allied health professions are influenced by multifactorial conditions including career development opportunities and workplace structures [11, 82].

Furthermore, the strong influence of social context and gendered perceptions suggests that targeted interventions are needed to broaden participation. Gender-sensitive recruitment strategies, increased visibility of diverse role models, and efforts to challenge stereotypical images of the professions [84, 85] (e.g., framing SLT as a purely female domain [49, 56], or reducing PT to fitness and massage [55, 63]) are essential to expand the pool of potential applicants. Addressing these stereotypes may also enhance perceived self-efficacy and outcome expectations, as described in SCCT [16, 24], thereby widening access to these professions. Overall, addressing workforce challenges in these fields will require both general structural support and tailored strategies that respect the unique identity of each profession [86, 87].

### Perspective on Germany

A key objective of this review was to focus on the German context. However, no studies were identified that systematically examine career choice processes in the OT, SLT, and PT professions within Germany. This gap raises the question of how well international findings can be transferred to the specific conditions of the German educational and healthcare systems.

Current labour market analyses demonstrate that OT, SLT, and PT are classified as bottleneck occupations in Germany, with severe shortages evident across federal states [88, 89]. The demographic transition further exacerbates these pressures, as rising demand from an ageing population outpaces the supply of new professionals [90]. While vocational training figures have seen slight increases, they fall short of meeting this workforce demand, underscoring ongoing structural challenges [91, 92].

Crucially, the findings of our review highlight that professional autonomy, clear career advancement opportunities, and financial stability are central determinants of career choice and retention. In Germany, these factors are currently the subject of intense structural debates and reforms. The country is in a complex transitional phase, expanding primary-qualifying university degree programmes alongside traditional vocational schools [93]. Professional alliances argue that strengthening academic pathways and expanding professional autonomy (e.g., through direct patient access) are essential to enhance professionalisation and long-term workforce sustainability [94, 95].

Our synthesis suggests that these current structural ambiguities may influence career decisions among prospective graduates by shaping their perceptions of job stability and professional status. While the recent abolition of tuition fees for vocational schools in many states has reduced financial hurdles [93], which was identified in our review as a crucial environmental support, profound structural challenges remain. High workloads, limited interprofessional parity, and restricted autonomy within the traditionally physician-led German healthcare system continue to impact the perceived attractiveness of these careers [96].

Consequently, the findings of this review have direct applied significance. They imply that to effectively address the recruitment crisis, policy initiatives must go beyond basic financial incentives and establish transparent career pathways that align with the expectations of contemporary students.

In summary, while findings related to personal factors (e.g., altruistic motivations) are likely universal and transferable across borders, the direct transfer of evidence regarding structural and professional factors is strictly limited. Therefore, contextual, empirically grounded evidence generated within Germany is urgently needed to inform policy, educational planning, and targeted recruitment efforts.

### Theoretical implications and future directions

The findings of this review demonstrate that career choice in the allied health professions is a multifaceted process, influenced by a dynamic interplay of personal, social, and structural factors. This underscores the need for integrative theoretical frameworks that can adequately model these complex interactions. Future research should therefore focus on developing empirical instruments to assess the key influencing factors identified in this review. Such instruments should be tailored to the German context by taking into account specific local features, such as the implications of primary qualifying degree programmes and direct access to therapy. Empirical studies based on these instruments would enable the development and validation of context-specific theoretical models and provide a robust evidence base for targeted interventions in career guidance and health policy.

### Limitations

This scoping review has several limitations that should be considered. First, the categorisation of influencing factors was based on an inductively developed coding scheme, which involves a degree of subjective judgment. To minimise bias, coding was performed by two independent reviewers, with discrepancies resolved through discussion and consensus. Second, the frequency with which a factor was mentioned was used as an indicator of its relevance. However, less frequently mentioned factors may still be highly significant in specific contexts or subpopulations. Third, in accordance with the established methodology for scoping reviews [26, 27], a formal quality appraisal of the included studies was not performed. While this is a defining feature of the scoping review approach, which prioritises breadth over depth, it means that the strength of evidence for individual factors was not critically assessed. Finally, the literature search was limited to publications in English and German. This language restriction may have introduced bias and could limit the generalisability of the findings to other linguistic or cultural contexts.

## Conclusions

This scoping review provides a comprehensive synthesis of the factors and theoretical frameworks influencing career choice in OT, SLT, and PT. The results indicate that the decision to pursue one of these allied health professions arises from a dynamic interaction between personal motivations and characteristics, social influences, and professional or structural conditions. While international literature offers valuable insights into these processes, the absence of empirical studies from Germany points to a substantial research gap. Addressing this gap requires context-specific investigations that reflect the unique features of the German educational and healthcare systems.

Based on these findings, there is a clear need for targeted, evidence-based strategies in career guidance, education, and policy to support the recruitment and retention of allied health professionals. Future research in the German context should focus on empirically validating theoretical models and examining how local structural and cultural factors shape career decision-making. Ultimately, a nuanced understanding of career choice processes is essential for ensuring the long-term stability and effectiveness of the allied health workforce and, consequently, the quality of healthcare delivery.

## Supplementary Information

Supplementary Material 1.

Supplementary Material 2.

Supplementary Material 3.

Supplementary Material 4.

## Data Availability

All data generated or analysed during this study are included in this published article and its supplementary information files.

## Abbreviations

OSF: Open Science Framework
OT: Occupational Therapy
PCC: Population, Concept, Context
PRISMA-ScR: Preferred Reporting Items for Systematic Reviews and Meta-Analyses Extension for Scoping Reviews
PT: Physiotherapy
SCCT: Social Cognitive Career Theory
SLT: Speech and Language Therapy
