# Supplementary material for: Career choice processes in occupational therapy, speech and language therapy, and physiotherapy: a scoping review
Source: BMC Med Educ. 2026 Apr 1;26:583. doi: 10.1186/s12909-026-09082-1 (PMC13064253; doi:10.1186/s12909-026-09082-1)
Supplement: Supplementary file 2 — Supplementary Material 2. [file 12909_2026_9082_MOESM2_ESM.docx]

**PubMed**

| 1 | "career choice*"[All Fields] OR "career pathway*"[All Fields] OR "occupational choice*"[All Fields] OR "career decision-making"[All Fields] OR "career intention*"[All Fields] OR "vocational interest*"[All Fields] OR "career aspiration*"[All Fields] OR "career development*"[All Fields] OR "vocational choice*"[All Fields] |
| --- | --- |
| 2 | "Speech Therapy"[MeSH Terms] OR "Occupational Therapy"[MeSH Terms] OR "Physical Therapists"[MeSH Terms] OR "occupational therap*"[All Fields] OR "physical therap*"[All Fields] OR "physiotherap*"[All Fields] OR "speech therap*"[All Fields] OR "speech patholog*"[All Fields] OR "logoped*"[All Fields] OR "SLT"[All Fields] OR "speech and language therap*"[All Fields] OR "allied health*"[All Fields] OR "health profession*"[All Fields] |
| 3 | "Sociological Factors"[MeSH Terms] OR "sociological factor*"[All Fields] OR "socioeconomic factor*"[All Fields] OR "cultural factor*"[All Fields] OR "cultural background"[All Fields] OR "cultural influence*"[All Fields] OR "social class"[All Fields] OR "social status"[All Fields] OR "socio demographic factor*"[All Fields] OR "demographic characteristic*"[All Fields] OR "personal value*"[All Fields] OR "self-concept"[All Fields] OR "self-perception"[All Fields] OR "personality trait*"[All Fields] OR "education pathway*"[All Fields] OR "educational background"[All Fields] OR "academic background"[All Fields] OR "schooling"[All Fields] OR "academic achievement*"[All Fields] OR "gender role*"[All Fields] OR "income"[All Fields] OR "social mobility"[All Fields] OR "motivation*"[All Fields] OR "intrinsic motivation*"[All Fields] OR "extrinsic motivation*"[All Fields] OR "career motivation*"[All Fields] OR "career decision self-efficacy"[All Fields] OR "career interest*"[All Fields] OR "educational aspiration*"[All Fields] OR "barrier*"[All Fields] OR "obstacle*"[All Fields] OR "challenge*"[All Fields] OR "facilitator*"[All Fields] OR "support network*"[All Fields] OR "social support*"[All Fields] OR "family influence*"[All Fields] |
| 4 | "theoretical model*"[All Fields] OR "theoretical framework*"[All Fields] OR "Person-Environment Fit Theory"[All Fields] OR "Self-Determination Theory"[All Fields] OR "Theory of Planned Behavior"[All Fields] OR "Rational Choice Theory"[All Fields] OR "career development theory"[All Fields] OR "social cognitive career theory"[All Fields] OR "Social Learning Theory"[All Fields] OR "RIASEC model"[All Fields] OR "Holland's Theory of Career Choice"[All Fields] OR "Status Attainment Theory"[All Fields] OR ("Circumscription and Compromise"[All Fields]) OR "Expectancy-Value Theory"[All Fields] OR “My System of Career Influnece*” [All Fields] OR "career decision-making model*"[All Fields] OR "Developmental Career Theory"[All Fields] OR ("career theory"[All Fields] OR "career theories"[All Fields]) |
| 5 | 1 AND 2 AND (3 OR 4) |

**Cochrane Central Register of Controlled Trials**

ID Search

#1 MeSH descriptor: [Career Choice] explode all trees

#2 career NEXT (choice* or pathway* or decision-making or intention* or aspiration* or development*)

#3 occupational NEXT choice*

#4 vocational (interest* or choice*)

#5 #1 OR #2 OR #3 OR #4

#6 MeSH descriptor: [Occupational Therapy] explode all trees

#7 MeSH descriptor: [Speech Therapy] explode all trees

#8 MeSH descriptor: [Physical Therapy Modalities] this term only

#9 occupational NEXT therap*

#10 physical NEXT therap*

#11 physiotherapy*

#12 speech NEXT therap*

#13 "speech and language" NEXT therap*

#14 allied NEXT health*

#15 health NEXT profession*

#16 #6 OR #7 OR #8 OR #9 OR #10 OR #11 OR #12 OR #13 OR #14 OR #15

#17 Sociological NEXT Factor*

#18 socioeconomic NEXT factor*

#19 cultural NEXT (factor* or background or influence*)

#20 social NEXT (class or status)

#21 socio NEXT demographic NEXT factor*

#22 demographic NEXT characteristic*

#23 personal NEXT value*

#24 self-concept

#25 self-perception

#26 personality NEXT trait*

#27 education NEXT pathway*

#28 educational NEXT background

#29 academic NEXT background

#30 schooling

#31 academic NEXT achievement*

#32 gender NEXT role*

#32 income

#33 social NEXT mobility

#34 motivation*

#35 intrinsic NEXT motivation*

#36 extrinsic NEXT motivation*

#37 career NEXT motivation*

#38 career NEXT decision NEXT self-efficacy

#39 career NEXT interest*

#40 educational NEXT aspiration*

#41 barrier*

#42 obstacle*

#43 challenge*

#44 facilitator*

#45 support NEXT network*

#46 social NEXT support*

#47 family NEXT influence*

#48 theoretical NEXT (model* or framework*)

#49 "Person Environment Fit Theory"

#50 "Self Determination Theory"

#51 "Theory of Planned Behavior"

#52 "Rational Choice Theory"

#53 "career development theory"

#54 "social cognitive career theory"

#55 "Social Learning Theory"

#56 "RIASEC model"

#57 "Holland's Theory of Career Choice"

#58 "Status Attainment Theory"

#59 "Circumscription and Compromise"

#60 "Expectancy Value Theory"

#61 career NEXT decision-making NEXT model*

#62 "Developmental Career Theory"

#63 career NEXT theor*

#64 "my system of career influences"

#65 #17 or #18 or #19 or #20 or #21 or #22 or #23 or #24 or #25 or #26 or #27 or #28 or #29 or #30 or #31 or #32 or #33 or #34 or #35 or #36 or #37 or #38 or #39 or #40 or #41 or #42 or #43 or #44 or #45 or #46 or #47 or #48 or #49 or #50 or #51 or #52 or #53 or #54 or #55 or #56 or #57 or #58 or #59 or #60 or #61 or #62 or #63 or #64

#66 #5 AND #16 AND #65

**CINAHL**

#1 "career choice*" OR "career pathway*" OR "occupational choice*" OR "career decision-making" OR "career intention*" OR "vocational interest*" OR "career aspiration*" OR "career development*" OR "vocational choice*" OR MM "Careers in Allied Health/CL/ED/EI/EV/HI/LJ/MT/PF/ST/TD/UT"

#2 “occupational therap*" OR "physical therap*" OR "physiotherap*" OR "speech therap*" OR "speech and language therap*" OR "allied health*" OR "health profession*" OR “SLT” OR “logoped*” OR “speech pathology*”

#3 "Sociological Factor*" OR "socioeconomic factor*" OR "cultural factor*" OR "cultural background" OR "cultural influence*" OR "social class" OR "social status" OR "socio demographic factor*" OR "demographic characteristic*" OR "personal value*" OR "self-concept" OR "self-perception" OR "personality trait*" OR "education pathway*" OR "educational background" OR "academic background" OR "schooling" OR "academic achievement*" OR "gender role*" OR "income" OR "social mobility" OR "motivation*" OR "intrinsic motivation*" OR "extrinsic motivation*" OR "career motivation*" OR "career decision self-efficacy" OR "career interest*" OR "educational aspiration*" OR "barrier*" OR "obstacle*" OR "challenge*" OR "facilitator*" OR "support network*" OR "social support*" OR "family influence*"

#4 "theoretical model*" OR "theoretical framework*" OR "Person-Environment Fit Theory" OR "Self-Determination Theory" OR "Theory of Planned Behavior" OR "Rational Choice Theory" OR "career development theory" OR "social cognitive career theory" OR "Social Learning Theory" OR "RIASEC model" OR "Holland's Theory of Career Choice" OR "Status Attainment Theory" OR "Circumscription and Compromise" OR "Expectancy-Value Theory" OR "my system of career influence*" OR "career decision-making model*" OR "Developmental Career Theory" OR "career theor*"

#1 AND #2 AND (#3 OR #4)

**Web of Science**

#1 ALL=("career choice*" OR "career pathway*" OR "occupational choice*" OR "career decision-making" OR "career intention*" OR "vocational interest*" OR "career aspiration*" OR "career development*" OR "vocational choice*" )

#2 ALL=(“occupational therap*" OR "physical therap*" OR "physiotherap*" OR "speech therap*" OR "speech and language therap*" OR "allied health*" OR "health profession*")

#3 ALL=("Sociological Factor*" OR "socioeconomic factor*" OR "cultural factor*" OR "cultural background" OR "cultural influence*" OR "social class" OR "social status" OR "socio demographic factor*" OR "demographic characteristic*" OR "personal value*" OR "self-concept" OR "self-perception" OR "personality trait*" OR "education pathway*" OR "educational background" OR "academic background" OR "schooling" OR "academic achievement*" OR "gender role*" OR "income" OR "social mobility" OR "motivation*" OR "intrinsic motivation*" OR "extrinsic motivation*" OR "career motivation*" OR "career decision self-efficacy" OR "career interest*" OR "educational aspiration*" OR "barrier*" OR "obstacle*" OR "challenge*" OR "facilitator*" OR "support network*" OR "social support*" OR "family influence*")

#4 ALL=("theoretical model*" OR "theoretical framework*" OR "Person-Environment Fit Theory" OR "Self-Determination Theory" OR "Theory of Planned Behavior" OR "Rational Choice Theory" OR "career development theory" OR "social cognitive career theory" OR "Social Learning Theory" OR "RIASEC model" OR “my system of career influence*” OR "Holland's Theory of Career Choice" OR "Status Attainment Theory" OR "Circumscription and Compromise" OR "Expectancy-Value Theory" OR "career decision-making model*" OR "Developmental Career Theory" OR "career theor*")

#1 AND #2 AND (#3 OR #4)

**Eric (Education Resources Information Center)**

("career choice" OR "career pathway" OR "occupational choice" OR "career decision-making" OR "career intention" OR "vocational interest" OR "career aspiration" OR "career development" OR "vocational choice")

AND

("occupational therapy" OR "physical therapy" OR "physiotherapy" OR "speech therapy" OR "speech and language therapy" OR “SLT” OR “logopedic” OR speech pathology” OR "allied health" OR "health profession")

AND

("Sociological Factor" OR "socioeconomic factor" OR "cultural factor" OR "cultural background" OR "cultural influence" OR "social class" OR "social status" OR "socio demographic factor" OR "demographic characteristic" OR "personal value" OR "self-concept" OR "self-perception" OR "personality trait" OR "education pathway" OR "educational background" OR "academic background" OR "schooling" OR "academic achievement" OR "gender role" OR "income" OR "social mobility" OR "motivation" OR "intrinsic motivation" OR "extrinsic motivation" OR "career motivation" OR "career decision self-efficacy" OR "career interest" OR "educational aspiration" OR "barrier" OR "obstacle" OR "challenge" OR "facilitator" OR "support network" OR "social support" OR "family influence" OR "theoretical model" OR "theoretical framework" OR "Person-Environment Fit Theory" OR "Self-Determination Theory" OR "Theory of Planned Behavior" OR "Rational Choice Theory" OR "career development theory" OR "social cognitive career theory" OR "Social Learning Theory" OR "RIASEC model" OR "Holland's Theory of Career Choice" OR "Status Attainment Theory" OR "Circumscription and Compromise" OR "Expectancy-Value Theory" OR "career decision-making model" OR "Developmental Career Theory" OR "career theory" OR “my system of career influences”)

**APA PsycInfo**

"career choice*" OR "career pathway*" OR "occupational choice*" OR "career decision-making" OR "career intention*" OR "vocational interest*" OR "career aspiration*" OR "career development*" OR "vocational choice*"

AND

"occupational therap*" OR "physical therap*" OR "physiotherapy*" OR "speech therap*" OR "speech and language therap*" OR "allied health*" OR "health profession*"

AND

"Sociological Factor*" OR "socioeconomic factor*" OR "cultural factor*" OR "cultural background" OR "cultural influence*" OR "social class" OR "social status" OR "socio demographic factor*" OR "demographic characteristic*" OR "personal value*" OR "self-concept" OR "self-perception" OR "personality trait*" OR "education pathway*" OR "educational background" OR "academic background" OR "schooling" OR "academic achievement*" OR "gender role*" OR "income" OR "social mobility" OR "motivation*" OR "intrinsic motivation" OR "extrinsic motivation" OR "career motivation*" OR "career decision self-efficacy" OR "career interest*" OR "educational aspiration*" OR "barrier*" OR "obstacle*" OR "challenge*" OR "facilitator*" OR "support network*" OR "social support*" OR "family influence*" OR "theoretical model*" OR "theoretical framework*" OR "Person-Environment Fit Theory" OR "Self-Determination Theory" OR "Theory of Planned Behavior" OR "Rational Choice Theory" OR "career development theory" OR "social cognitive career theory" OR "Social Learning Theory" OR "RIASEC model" OR "Holland's Theory of Career Choice" OR "my system of career influence*" OR "Status Attainment Theory" OR "Circumscription and Compromise" OR "Expectancy-Value Theory" OR "career decision-making model*" OR "Developmental Career Theory" OR "career theor*"

**German-Language Grey Literature and Web Search Strategy**

To identify relevant literature specific to the German context, targeted searches were conducted in December 2024. Due to the limitations of search interfaces on institutional websites, simplified combinations of the following German keywords were used:

- **Target Professions:** *Ergotherapie, Logopädie, Physiotherapie, Therapieberufe, Gesundheitsfachberufe*
- **Target Concepts:** *Berufswahl, Studienwahl, Motivation, Einflussfaktoren, Berufsorientierung, Nachwuchs, Fachkräftemangel*

**Searched Sources:**
Searches were conducted across the following relevant German institutional repositories and professional association websites:

**Professional Associations:**

- - Deutscher Verband Ergotherapie e.V. (DVE)
  - Deutscher Bundesverband für Logopädie e.V. (dbl)
  - Deutscher Verband für Physiotherapie (ZVK) e.V. (Physio-Deutschland)
  - Hochschulverbund Gesundheitsfachberufe e.V. (HVG)

**Institutional and Governmental Repositories:**

- - Bundesinstitut für Berufsbildung (BIBB) - VET Repository und Bibliothekskatalog
  - Wissenschaftsrat (WR) - Publikationen
